# Glycosylation converts CD24 from a developmental marker into a glyco-immune checkpoint in B-cell acute lymphoblastic leukemia

**DOI:** 10.3389/fimmu.2026.1952918

**Published:** 2026-09-16

**Authors:** Shuheng Yan, Xuehong Zhang

**Affiliations:** 1 Center of Genome and Personalized Medicine, Institute of Cancer Stem Cell, Dalian Medical University, Dalian, China; 2 Department of Translational Genomics, Keck School of Medicine of the University of Southern California, Los Angeles, CA, United States

**Keywords:** B-cell acute lymphoblastic leukemia, CD24, glycosylation, innate immune checkpoint, leukemia glycoimmunology, Siglec-10

## Abstract

CD24 is widely recognized as a developmental marker of early B-cell differentiation and is highly expressed in precursor B-cell acute lymphoblastic leukemia (B-ALL). However, CD24 expression alone does not fully explain its biological activity, suggesting that post-translational regulation, particularly glycosylation, may determine its immune-regulatory functions. Although the CD24–Siglec-10 axis has emerged as an important innate immune checkpoint, how developmental CD24 expression is functionally adapted during leukemogenesis remains poorly understood. Here, we propose a framework in which CD24 functions as a glyco-immune checkpoint whose activity is determined by functional glycoforms rather than protein abundance alone. We discuss how physiological CD24 expression during B-cell ontogeny may contribute to developmental immune tolerance and propose that this program can be retained, hijacked, or remodeled during leukemogenesis through altered glycosylation patterns. Such glyco-adaptation may enhance CD24–Siglec-10-mediated suppression of macrophage surveillance, allowing leukemia cells to evade innate immune recognition and persist as minimal residual disease. We further propose that glycosylation-dependent immune adaptation represents an additional layer of leukemia evolution that complements genetic and epigenetic alterations. This perspective integrates developmental biology, glycobiology, innate immunity, and leukemia biology to establish the emerging field of leukemia glycoimmunology. Finally, we discuss the translational implications of CD24 glycobiology, including glycoform-based biomarkers, functional immune profiling, glycosylation-directed therapeutic strategies, and modulation of glycan remodeling pathways. Understanding how glycosylation shapes CD24 function may provide new opportunities for precision immunotherapy in B-ALL.

## 1 Introduction

During normal B-cell ontogeny, hematopoietic stem cells progressively differentiate through a series of lymphoid developmental intermediates, including multipotent progenitors, lymphoid-primed progenitors, and common lymphoid progenitors, before giving rise to immature and mature B cells (1–3). Throughout this developmental trajectory, CD24 expression is tightly regulated in a stage-dependent manner (4, 5). CD24 is minimally expressed in early hematopoietic stem and progenitor compartments, emerges after lymphoid commitment, increases during early B-cell differentiation, reaches its highest levels at the pre-B and immature B-cell stages, and subsequently declines during terminal maturation (6, 7). This developmental expression pattern suggests that CD24 is not simply a lineage-associated marker but represents an acquired component of the B-cell maturation program. The preferential expression of CD24 during early B-cell development, particularly at the pre-B-cell stage, coincides with a period of active proliferation, receptor rearrangement, and developmental selection, raising the possibility that CD24 contributes to the coordination of B-cell differentiation with local immune homeostasis (7). This developmental expression pattern provides a framework for understanding the biological significance of CD24 in B-cell acute lymphoblastic leukemia (B-ALL). We propose that the key transition during leukemogenesis is not simply the persistence of CD24 expression, but the acquisition of functional properties through glycosylation-dependent remodeling. Rather than acting solely as a retained developmental marker, CD24 glycoforms may acquire distinct immune-regulatory functions by engaging inhibitory pathways such as the CD24–Siglec-10 axis. This perspective highlights how developmental programs can be functionally adapted during leukemogenesis and provides a basis for understanding innate immune escape, minimal residual disease (MRD) persistence, and leukemia evolution.

This Review presents a conceptual framework integrating developmental biology, glycobiology, and innate immunity to generate testable hypotheses regarding CD24 biology in B-ALL. Unless otherwise stated, several proposed models discussed herein remain hypothetical and require future experimental validation.

## 2 CD24: more than a developmental marker

For decades, CD24 has been regarded primarily as a differentiation antigen rather than a functional regulator (8–10). In hematology, CD24 has been widely used as a component of multiparameter flow cytometry panels to define B-cell developmental stages and support the immunophenotypic diagnosis of precursor B-ALL (11, 12).

Given its high expression during normal B-cell maturation and its frequent retention in B-ALL blasts, CD24 has traditionally been interpreted as a lineage-associated marker reflecting developmental identity (5, 9, 13). Consequently, previous studies have largely focused on its expression patterns, diagnostic utility, and prognostic implications, whereas its functional contribution to leukemia biology remained comparatively unexplored (6, 14, 15).

Recent advances have substantially expanded this view by demonstrating that CD24 is not merely a passive marker of cellular identity but an active regulator of innate immune responses (15–17). Through interaction with Siglec-10 expressed on macrophages and other myeloid cells, CD24 can suppress inflammatory signaling, modulate phagocytic activity, and contribute to immune tolerance (18, 19). These findings established the CD24–Siglec-10 axis as an innate immune checkpoint and positioned CD24 within the broader framework of tumor immune regulation (15–17, 19–21). Importantly, this emerging perspective suggests that the biological significance of CD24 extends beyond lineage specification and may be determined by its ability to regulate immune surveillance (19–21). This raises a fundamental question: how is a developmentally regulated surface molecule functionally adapted during leukemogenesis?

At the same time, emerging evidences have revealed the important roles of post-translational glycosylation in immune checkpoint proteins (15, 22). Because Siglec-10 preferentially recognizes glycan structures rather than the peptide backbone of CD24, alterations in CD24 glycosylation may modulate its immune-regulatory activity without substantially affecting overall expression levels (23–25). This concept challenges the conventional assumption that CD24 expression directly reflects biological function and highlights the distinction between quantitative expression and qualitative functional state (22–25).

This distinction is particularly relevant to B-ALL, a malignancy that arises from precursor B cells naturally expressing high levels of CD24. A key unresolved question is whether leukemic CD24 simply reflects the retention of a normal developmental program or undergoes functional adaptation to support immune evasion. We propose that the transition from developmental expression to functional remodeling represents an important conceptual framework for understanding CD24 biology during B-ALL evolution.

Thus, CD24 should be viewed not merely as a marker of B-cell differentiation, but as a dynamic interface connecting developmental programs, glycosylation-dependent regulation, innate immunity, and leukemia evolution. This perspective provides the foundation for exploring CD24 as a developmentally programmed glyco-immune checkpoint and frames the subsequent discussion of how glycosylation may shape CD24 function in B-ALL.

## 3 Developmental expression of CD24 during B-cell ontogeny: a developmental program beyond lineage commitment

B-cell development represents a highly coordinated differentiation process in which progenitor cells progressively acquire lineage identity, functional competence, and immune regulatory properties (26–28). During this developmental trajectory, regulators such as CD24 undergo stage-specific expression changes that accompany distinct maturation states (6). CD24 expression is low or absent in early hematopoietic compartments, emerges following B-lineage commitment, increases during early B-cell differentiation, reaches high levels at the pre-B and immature B-cell stages, and declines during terminal maturation (6). This dynamic expression pattern suggests that CD24 is not merely a marker of B-cell differentiation but represents a developmentally regulated program associated with specific stages of lymphoid maturation.

The pre-B-cell stage represents a critical checkpoint during which precursor B cells undergo extensive proliferation, immunoglobulin rearrangement, and developmental selection (29, 30). These processes require coordinated regulation of differentiation, survival, and immune homeostasis. Emerging evidence suggests that CD24 may contribute to this developmental balance by participating in mechanisms that limit excessive innate immune activation. Through interaction with Siglec-G in mice and its human orthologue Siglec-10, CD24 functions as a negative regulator of inflammatory responses triggered by endogenous danger-associated molecular patterns (DAMPs) (18, 31). Although direct evidence linking CD24 to immune tolerance during human B-cell ontogeny remains limited, we propose that abundant CD24 expression during early B-cell development may provide a protective context that supports orderly maturation within the bone marrow niche while preventing inappropriate immune-mediated elimination.

Based on this concept, we propose the term developmental immune tolerance, referring to a physiological process in which immune-regulatory molecules coordinate lymphoid development with local immune homeostasis. In this framework, CD24 extends beyond a differentiation marker and represents a component of the developmental machinery that integrates B-cell maturation with immune regulation. This perspective may explain why high CD24 expression is retained in precursor B-ALL and raises an important question: does leukemic transformation simply preserve a developmental CD24 program, or does it functionally adapt this program to promote immune escape? Addressing this question provides the conceptual foundation for understanding how CD24 transitions from a developmental regulator to a glyco-immune checkpoint in B-ALL.

## 4 Retention, hijacking, or remodeling? Three conceptual models of CD24 biology in B-cell acute lymphoblastic leukemia

The high expression of CD24 in B-ALL has traditionally been interpreted as a consequence of its precursor B-cell origin, reflecting the retention of a normal developmental phenotype rather than an actively regulated biological program (5, 12–14). However, emerging insights from tumor immunology and glycobiology indicate that immune-regulatory functions are often shaped by post-translational modifications and molecular context rather than protein abundance alone (32–34). Therefore, we suspect that CD24 expression in B-ALL may represent multiple, non-mutually exclusive biological states rather than a single developmental outcome.

Here, we propose a conceptual framework comprising three non-mutually exclusive models that leukemogenesis may retain, hijack, or remodel the CD24 developmental program, linking B-cell maturation with innate immune regulation and leukemia immune adaptation. These three conceptual models provide a framework for understanding how a developmentally regulated molecule may acquire distinct biological roles during B-ALL evolution (**Figure 1**).

**Figure 1:**
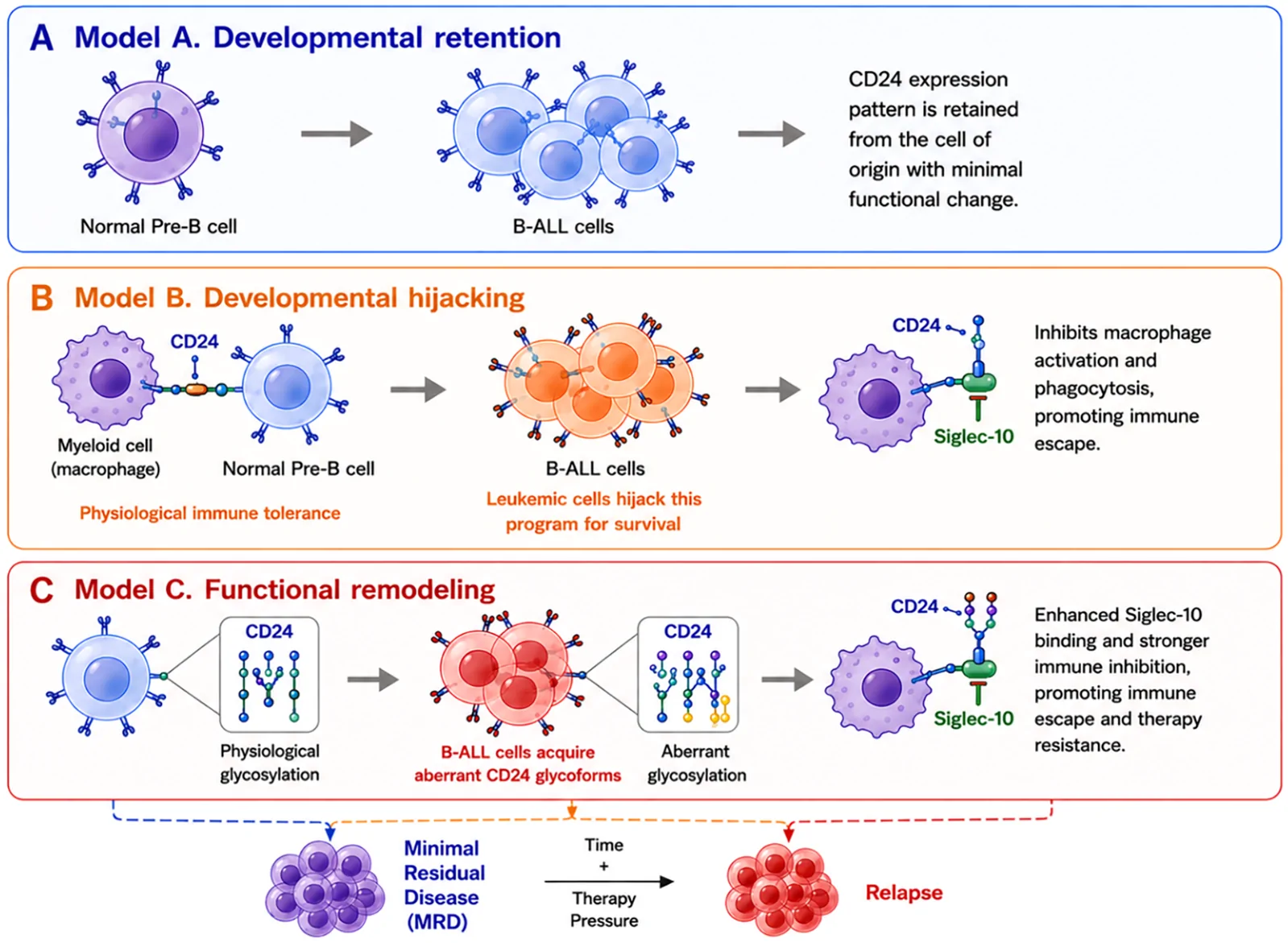
A conceptual framework illustrating three proposed models may explain the biological significance of CD24 in B-cell acute lymphoblastic leukemia. **(A)** Developmental retention (proposed). The physiological CD24 expression program established during normal pre-B-cell development is retained after leukemic transformation with minimal functional alteration. In this model, CD24 expression primarily reflects the developmental origin of B-ALL. **(B)** Developmental hijacking (proposed). Leukemic cells exploit the physiological CD24-mediated immune tolerance program to suppress macrophage activation through the CD24–Siglec-10 axis, thereby promoting innate immune escape and providing a survival advantage within the bone marrow microenvironment. **(C)** Functional remodeling (proposed). Beyond retaining developmental CD24 expression, leukemic cells undergo glycosylation remodeling, generating functionally distinct CD24 glycoforms with enhanced Siglec-10 binding and stronger immune-inhibitory activity. This qualitative remodeling promotes immune evasion, facilitates persistence under therapeutic pressure, and contributes to minimal residual disease (MRD) and eventual relapse. These models are not mutually exclusive and are intended to provide a conceptual framework for future experimental validation rather than definitive biological mechanisms.

### 4.1 Model A. Developmental retention: preservation of physiological CD24 expression

The first conceptual model proposes that leukemic transformation primarily preserves the physiological CD24 expression pattern of precursor B cells. In this context, high CD24 expression reflects the developmental stage at which leukemic blasts are arrested, as precursor B cells naturally express abundant CD24 during normal maturation (6, 11, 12). Under this model, CD24 mainly represents a marker of developmental identity, and its immune-regulatory activity may reflect the physiological functions associated with this developmental stage rather than a leukemia-specific adaptation. This speculative model is consistent with the widespread expression of CD24 across precursor B-ALL subtypes and emphasizes the developmental origin of CD24 positivity in leukemia.

### 4.2 Model B. Developmental hijacking: co-option of physiological immune tolerance

A second conceptual model proposes that leukemic cells actively exploit a physiological immune-regulatory program that normally supports precursor B-cell development. During normal lymphopoiesis, high CD24 expression may contribute to developmental immune tolerance by limiting excessive innate immune activation through interaction with Siglec-G in mice and Siglec-10 in humans (15–18, 35). Although direct evidence in human B-cell development remains limited, B-ALL cells may preserve or amplify this immune-protective state to reduce macrophage-mediated immune surveillance within the bone marrow microenvironment. In this model, we propose that CD24 is not merely retained but may be functionally adapted to support leukemia immune evasion.

### 4.3 Model C. Functional remodeling: glycosylation may functionally reshape the immune function of CD24

The third conceptual model proposes that leukemic transformation may alter CD24 function through glycosylation-dependent remodeling. Although CD24 surface expression may remain comparable between normal precursor B cells and leukemic blasts, changes in glycan composition may generate functionally distinct CD24 glycoforms with altered interaction with Siglec-10 and differential immune-regulatory activity (22, 34–36). Under this model, the biological function of CD24 is determined not simply by expression level but by its glycosylation-defined molecular state (7, 37–40). This perspective shifts the focus from quantitative expression to functional glycobiology and provides a potential mechanism by which leukemia cells acquire enhanced immune escape capacity, persistence as MRD, and relapse potential.

These conceptual models should not be viewed as mutually exclusive mechanisms. Instead, we propose that they may represent different stages or overlapping states during leukemia evolution. Developmental retention provides the initial context in which CD24 is expressed, developmental hijacking may exploit its physiological immune-regulatory properties, and glycosylation-dependent remodeling may further refine CD24 function under selective pressures imposed by the immune microenvironment and therapy.

At present, direct evidence distinguishing these possibilities remains limited. Comparative analyses of CD24 glycosylation and function across normal precursor B cells, newly diagnosed B-ALL, MRD, and relapsed leukemia will be required to determine how CD24 evolves during leukemogenesis. Future studies should therefore move beyond measuring CD24 abundance and investigate how developmental programs are functionally adapted through glycobiology during leukemia progression (41–43).

## 5 From protein expression to functional glycoforms: functional glycobiology determines CD24 immune activity

A central principle emerging from recent studies is that CD24 function cannot be inferred solely from its expression level (37, 38). Although CD24 has traditionally been quantified by flow cytometry or immunohistochemistry as a surface marker of B-cell differentiation, accumulating evidence indicates that its immune-regulatory activity is shaped largely by post-translational glycosylation and the resulting molecular context rather than by protein abundance alone (25, 39, 40).

CD24 is a small glycosylphosphatidylinositol (GPI)-anchored membrane protein with extensive N- and O-linked glycosylation, and these glycan structures account for much of its biochemical complexity (10, 28, 44, 45). Even the super-heterogeneous CD24-Fc fusion protein, developed as a glycosylated biotherapeutic, has been found to exist in over 300 distinct glycoforms (46). In the context of cancer, dysregulated glycosyltransferase activity and extensive glycome remodeling can modulate the abundance, membrane localization, and cell surface binding of the CD24 protein (7, 37, 38, 47, 48). Thus, the biological identity of CD24 is determined not only by how much CD24 is expressed, but also by the molecular features of its glycan architecture (**Figure 2A**).

**Figure 2:**
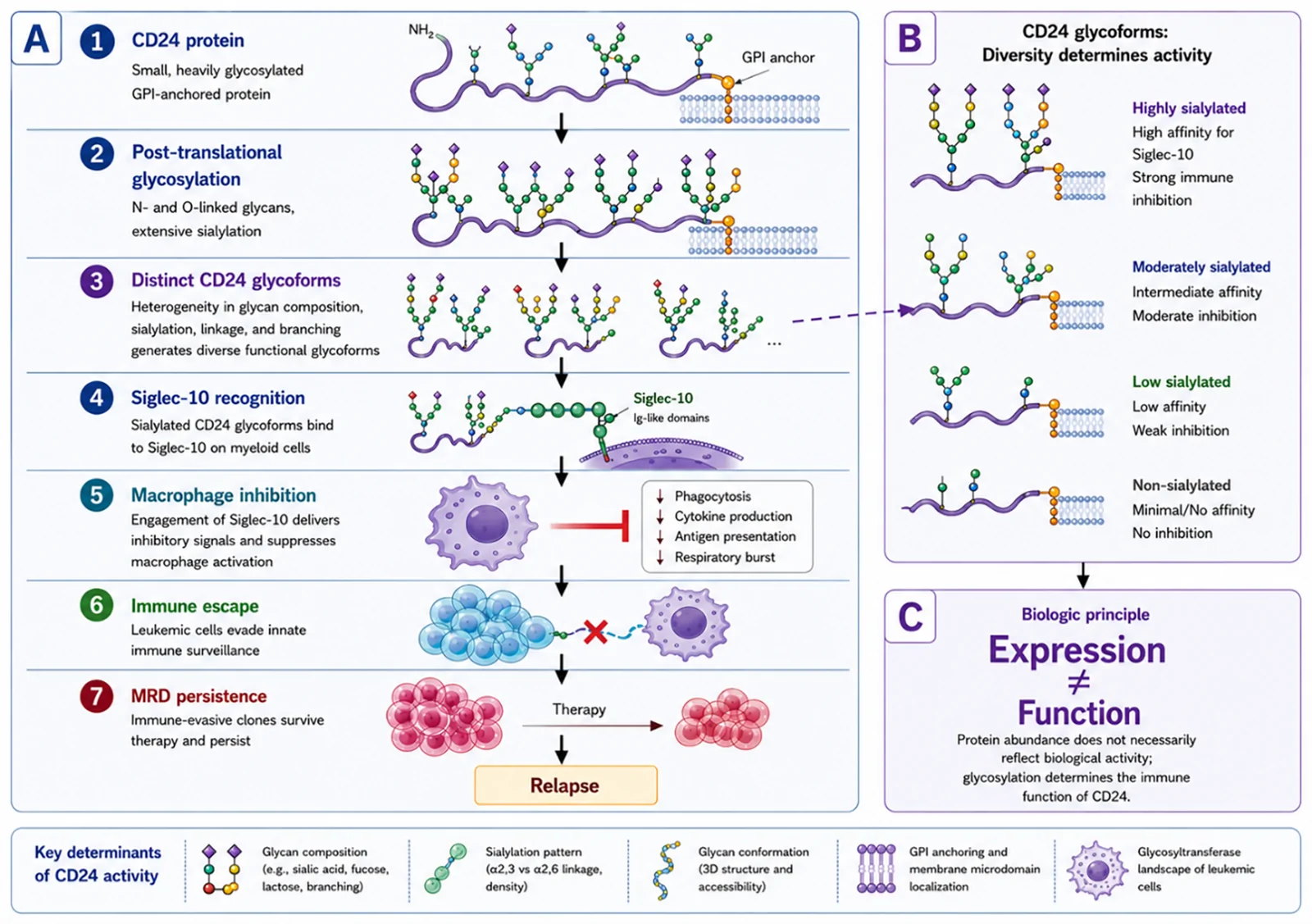
Proposed functional glycobiology of CD24: glycosylation determines immune activity rather than protein abundance. **(A)** Proposed stepwise model of functional CD24 glycobiology. 1) CD24 protein. CD24 is a small, heavily glycosylated glycosylphosphatidylinositol (GPI)-anchored membrane protein. 2) Post-translational glycosylation. Extensive N- and O-linked glycosylation generates the mature functional form of CD24. 3) Distinct CD24 glycoforms. Differences in glycan composition, sialylation, branching, and linkage produce structurally and functionally distinct CD24 glycoforms. 4) Siglec-10 recognition. Sialylated CD24 glycoforms preferentially bind Siglec-10 on myeloid cells, initiating inhibitory signaling. 5) Macrophage inhibition. CD24–Siglec-10 signaling suppresses macrophage activation, phagocytosis, and inflammatory responses. 6) Innate immune escape. Functionally active CD24 glycoforms facilitate innate immune evasion through enhanced Siglec-10-mediated inhibition. 7) MRD persistence and relapse. Immune-selected leukemic cells persist after therapy, promoting MRD maintenance and disease relapse. **(B)** Proposed functional diversity of CD24 glycoforms. Increasing levels of sialylation and specific glycan architectures enhance Siglec-10 binding affinity and inhibitory signaling, whereas poorly sialylated or non-sialylated glycoforms exhibit limited immune-regulatory activity. Thus, structurally distinct glycoforms display markedly different biological functions despite similar CD24 protein expression. **(C)** Central biological principle proposed in this Mini-Review. CD24 expression does not necessarily predict CD24 function. Instead, its immune-regulatory activity is determined primarily by glycosylation, making functional CD24 glycoforms—rather than protein abundance—the key molecular unit governing CD24-mediated innate immune regulation.

Among these modifications, terminal sialylation represents a critical determinant of CD24-mediated immune regulation. The human CD24 receptor Siglec-10 recognizes sialylated glycan structures associated with CD24 rather than the peptide backbone itself, and this interaction is influenced by glycan composition, density, linkage, and spatial organization (35, 49, 50). Consequently, alterations in glycosyltransferase activity, sialylation patterns, or glycan branching may modify CD24 immune activity without altering total CD24 expression.

This glycosylation-centered perspective has important implications for B-ALL biology. Leukemic samples with comparable levels of CD24 surface expression may contain distinct glycoform landscapes and therefore differ in their ability to engage Siglec-10, regulate macrophage activity, and evade innate immune surveillance. Conventional measurement of CD24 abundance may therefore provide an incomplete representation of its functional state. Instead, the biological activity of CD24 is likely determined by the qualitative properties of its glycoforms (**Figure 2B**).

Beyond macrophages, murine Siglec-G and its human orthologs Siglec-10 are also expressed on B cells, where they function as negative regulators of B-cell receptor (BCR) signaling (51–53). This localization raises the intriguing possibility that CD24 and Siglec-10 might engage in *cis* interactions on the surface of B-ALL cells. While one study has reported the downmodulation of Siglec-10 on human chronic lymphocytic leukemia (CLL) cells (54), direct evidence for Siglec-10 expression in B-ALL remains elusive. In the murine Eμ-TCL1 model, Siglec-G deficiency drives earlier onset and more severe CLL-like disease, whereas its overexpression confers almost complete protection (54). Thus, whether CD24 and Siglec-10 indeed interact in *cis* on B-ALL cells is still unknown. Even if such an interaction does occur, several critical questions remain: (i) whether its inhibitory effect on BCR signaling contributes to B-ALL pathogenesis, (ii) whether the *cis* engagement itself induces immunosuppression, and (iii) in the presence of both *cis* and *trans* interactions, how the *cis* engagement might modulate the immunosuppressive function of the *trans* interaction. In addition, the potential effects of varying Siglec-10 levels and subcellular distribution on these processes also warrants investigation. Future studies integrating glycoproteomics, receptor interaction analyses, and functional assays will be essential to definitively address these questions and to clarify the role of this pathway in leukemogenesis and immune adaptation.

This concept shifts the study of CD24 from expression-based classification toward functional glycobiology. In this framework, CD24 should be viewed not as a static differentiation antigen, but as a dynamic glycoprotein whose immune-regulatory properties are continuously shaped by glycan remodeling. Leukemic cells may therefore preserve developmental CD24 expression while acquiring enhanced immune-evasive capacity through glycosylation-dependent functional adaptation.

From a translational perspective, this model raises several important questions: whether distinct CD24 glycoforms characterize newly diagnosed, minimal residual disease, and relapsed B-ALL; whether therapeutic pressure induces glycosylation remodeling independently of CD24 expression; and whether glycoform-specific antibodies, lectin-based strategies, or single-cell glycoproteomic approaches can identify functionally active CD24 populations beyond conventional immunophenotyping (41–43, 55).

Together, these concepts establish a central principle of CD24 biology: CD24 expression does not necessarily predict CD24 function (**Figure 2C**). Rather, glycosylation likely defines the functional state of CD24 and determines its capacity to regulate innate immune responses.

## 6 CD24 represents a prototype glyco-immune checkpoint

The identification of the CD24–Siglec-10 axis has expanded the concept of immune checkpoint biology beyond conventional protein–protein interactions (7, 20, 56). Unlike classical immune checkpoints, including PD-1, PD-L1, and CTLA-4, CD24 regulates immune responses through glycan-dependent recognition by Siglec-10, a sialic acid-binding inhibitory receptor expressed on macrophages and other myeloid cells (7, 50, 56). This distinct mode of immune regulation defines CD24 as an emerging class of glycan-dependent immune checkpoints.

Upon engagement with functional sialylated CD24 glycoforms, Siglec-10 transmits inhibitory signals through intracellular immunoreceptor tyrosine-based inhibitory motifs (ITIMs), leading to recruitment of phosphatases such as SHP-1 and SHP-2 (50, 57). This signaling pathway restrains macrophage activation, phagocytic activity, and inflammatory cytokine production, thereby contributing to immune homeostasis by limiting excessive responses to endogenous danger-associated signals (15, 19, 20, 35).

During tumor development, however, this physiological immune-regulatory mechanism may be exploited by malignant cells. Tumor-associated CD24 glycoforms can engage Siglec-10 on macrophages, generating inhibitory signals that reduce macrophage-mediated clearance and promote immune escape independently of adaptive immune checkpoints such as PD-1 or CTLA-4 (15). Thus, CD24 introduces an additional layer of immune regulation based on glycan recognition rather than conventional receptor–ligand interactions.

Importantly, CD24 should not be viewed simply as a protein-based immune checkpoint. Its immune-regulatory activity is determined by the glycosylation landscape that governs receptor recognition and downstream signaling, including glycan composition, terminal sialylation, branching patterns, and spatial organization (7, 50). Consequently, alterations in glycosylation may reshape CD24 activity without changes in overall surface expression.

This concept is particularly relevant to B-ALL, in which precursor B-cell-derived blasts frequently retain high levels of CD24 expression (6, 10, 13). We propose that leukemic cells may preserve or functionally adapt this developmental program by acquiring CD24 glycoforms capable of engaging Siglec-10, reducing macrophage surveillance, and promoting immune tolerance within the bone marrow microenvironment. Such glyco-immune adaptation may contribute to leukemia persistence and MRD despite stable CD24 expression.

Collectively, these findings position CD24 as a prototype glyco-immune checkpoint, in which glycosylation, rather than protein abundance alone, determines immune-regulatory activity. This concept expands the immune checkpoint paradigm beyond protein-centered interactions and establishes a conceptual link between glycobiology, innate immunity, and leukemia evolution. It also suggests that future therapeutic strategies may require targeting functional CD24 glycoforms or glycosylation-dependent pathways rather than CD24 expression alone.

## 7 Functional glycobiology of MRD: from drug resistance to immune-selected persistence

MRD represents the strongest predictor of relapse in B-ALL (14, 58–61). Although persistent leukemia cells have traditionally been interpreted as a consequence of intrinsic resistance mechanisms, including genetic evolution, cellular quiescence, altered apoptosis, and protective microenvironmental interactions (62–65), these mechanisms alone may not fully explain the long-term survival of residual clones after therapy.

We propose that MRD should be viewed not only as a drug-resistant population but also as an immune-selected reservoir. During treatment, residual leukemia cells are continuously exposed to selective pressures imposed by both therapy and the host immune microenvironment. Under these conditions, cellular states capable of establishing effective immune tolerance may be preferentially maintained. CD24 glycosylation remodeling represents a potential mechanism through which residual leukemia cells adapt to innate immune surveillance.

Importantly, therapeutic selection may not simply favor leukemia cells with higher CD24 expression. Instead, it may select for populations carrying functionally distinct CD24 glycoforms with enhanced capacity to engage Siglec-10 and suppress macrophage-mediated clearance (7, 15, 47). In this context, the biologically relevant determinant is not CD24 abundance itself, but the glycosylation-defined functional state of CD24.

Based on this concept, we introduce the term glycoediting, referring to the immune-driven selection and remodeling of glycosylation patterns that confer enhanced immune-regulatory properties. Analogous to cancer immunoediting (66–68), glycoediting represents a dynamic process in which selective immune pressure may progressively shape leukemic populations toward states that evade innate immune recognition. Through this process, CD24 glycoforms may contribute to MRD persistence and subsequent relapse.

This model predicts that CD24 glycoforms, rather than total CD24 expression, may serve as functional biomarkers of immune adaptation. Longitudinal characterization of CD24 glycosylation during treatment may identify immune-selected leukemia populations and reveal new therapeutic vulnerabilities. Collectively, we propose a revised framework for understanding MRD biology: MRD is not merely a reservoir of therapy-resistant cells but may represent an immune-selected population shaped by glycoediting. In this framework, CD24 glycoforms function both as mediators of immune escape and as potential targets for preventing leukemia persistence and relapse.

## 8 Translational opportunities arising from functional glycobiology

Recognition of CD24 as a glycosylation-dependent innate immune checkpoint expands its therapeutic potential beyond conventional antigen-based targeting strategies (7, 15). Because CD24 protein abundance does not fully capture its immune-regulatory activity, future approaches should focus on defining, monitoring, and therapeutically targeting functional CD24 glycoforms in B-ALL.

### 8.1 CD24 glycoforms as biomarkers of immune adaptation

Functional CD24 glycoforms may provide biomarkers that more accurately reflect leukemia immune adaptation, MRD persistence, and relapse risk than total CD24 expression alone. Glycoform-specific detection strategies, combined with glycoproteomic and single-cell approaches, may identify biologically active CD24 states and complement current MRD monitoring by revealing whether residual leukemia has acquired immune-evasive properties.

### 8.2 Functional assessment of CD24 immune activity

Because CD24 checkpoint activity depends on glycan-mediated recognition by Siglec-10, functional assays evaluating Siglec-10 engagement, macrophage activation, and phagocytic responses may provide direct measurements of CD24-mediated immune regulation (25, 37). Such approaches may enable identification of leukemia subsets that preferentially rely on CD24-dependent innate immune escape and facilitate patient stratification.

### 8.3 Glycoform-directed immunotherapy

The recognition that CD24 function is determined by glycoform composition rather than protein abundance suggests a shift from targeting total CD24 toward selectively targeting leukemia-associated CD24 states. Glycoform-specific antibodies, glycan-directed therapeutic strategies, and emerging glycoform-selective CAR-based approaches may improve tumor specificity by distinguishing malignant CD24 glycoforms from physiological CD24 expression during normal B-cell development (69–72). Combining CD24 glyco-immune targeting with existing immunotherapies, including CAR-T cell approaches, may provide complementary strategies to overcome both innate and adaptive immune escape mechanisms (73–75).

### 8.4 Targeting glycosylation remodeling to prevent immune escape

Beyond direct targeting of CD24, modulation of glycosylation pathways represents an emerging strategy to interfere with the generation of immune-evasive CD24 glycoforms. Targeting glycoediting processes that shape immune-selected leukemia populations may provide an alternative approach to restore immune recognition and enhance therapeutic responses.

Together, these translational opportunities support a shift from measuring CD24 expression toward defining CD24 functional states. Future precision approaches in B-ALL may require integration of antigen abundance, glycoform composition, and immune functional activity to identify and target leukemia populations driven by glyco-immune adaptation.

## 9 Perspective and conclusion

### 9.1 Beyond CD24: a new framework for leukemia glycoimmunology

For decades, leukemia evolution has been primarily interpreted through a genome-centered paradigm, in which genetic alterations drive disease initiation, clonal selection, therapeutic resistance, and relapse (76–78). This framework has fundamentally transformed precision hematology; however, genetic evolution alone may not fully capture the biological complexity underlying leukemia heterogeneity, MRD persistence, and the dynamic interaction between leukemic cells and the immune microenvironment (79, 80).

Here, we propose that leukemia evolution involves an additional layer of biological adaptation beyond genetic change: glycosylation remodeling. Unlike the relatively stable genome, the glycome represents a highly dynamic and context-dependent system shaped by inflammatory signals, metabolic states, therapeutic pressure, and microenvironmental selection (7, 35, 38, 44, 45, 47, 48). This plasticity may allow leukemic cells to rapidly adapt to immune constraints through reversible alterations in cell-surface glycan composition without requiring additional genetic alterations.

CD24 provides a conceptual example of this emerging principle. As discussed throughout this review, CD24 should not be viewed simply as a developmental marker, but as a prototype glyco-immune checkpoint whose immune-regulatory activity is determined by functional glycoforms rather than protein abundance alone (7, 15, 22, 25). We propose that leukemic evolution is driven not only by genetic selection but also by glyco-functional adaptation, whereby immune pressure may favor leukemic states capable of engaging innate immune checkpoints, suppressing macrophage surveillance, and maintaining disease persistence.

This perspective expands the classical model of clonal evolution by incorporating glyco-immune adaptation as an additional evolutionary dimension. In this framework, glycosylation remodeling may do not replace genetic evolution but function as a complementary mechanism that shapes the interaction between leukemia cells and their immune ecosystem (7, 15, 35, 48). Beyond CD24 and B-ALL, glycosylation-dependent regulation of immune receptors, adhesion molecules, and checkpoint pathways suggests that glyco-remodeling may represent a broader mechanism of immune adaptation across hematologic malignancies. Future integration of glycoproteomics, spatial glycomics, single-cell multi-omics, and functional immunology will be essential to determine how glycosylation states evolve during leukemia progression and therapy. Such approaches may establish leukemia glycoimmunology as an emerging field connecting glycobiology, tumor immunology, and precision hematology (41–43, 49, 50, 52, 55).

We emphasize that the models proposed in this Review are intended to provide a conceptual framework for future investigation rather than definitive mechanistic conclusions. Their validation will require integrated analyses of glycoproteomics, glycan structural profiling, quantitative Siglec-10 binding, macrophage functional assays, and longitudinal patient samples spanning diagnosis, MRD, and relapse. Such studies will determine whether glycosylation remodeling functionally reshapes CD24-mediated innate immune regulation during B-ALL evolution and establish the biological and translational significance of functional glycobiology.

Ultimately, CD24 represents not an endpoint, but an entry point into a broader biological framework. Understanding how glycosylation shapes immune recognition and cellular adaptation may redefine current concepts of leukemia evolution and reveal new opportunities for biomarker discovery, immune monitoring, and therapeutic intervention. The future of leukemia biology may therefore move toward an integrated evolutionary model in which genetic alterations and glycosylation remodeling act as complementary forces shaping disease progression, immune adaptation, and treatment response.
